# Screening and characterization estrogen receptor ligands from *Arnebia euchroma* (Royle) Johnst. *via* affinity ultrafiltration LC-MS and molecular docking

**DOI:** 10.3389/fpls.2022.1012553

**Published:** 2022-11-07

**Authors:** Lian Zhu, Sheng-jun Ma, Ming-juan Liu, Kai-lin Li, Shuai E, Zi-ming Wang, Sha-ni Li, Sheng-lan Zhang, Wei Cai

**Affiliations:** ^1^ College of Food Science and Pharmacy, Xinjiang Agricultural University, Urumqi, China; ^2^ School of Pharmaceutical Sciences, Sino-Pakistan Center on Traditional Chinese Medicine, Hunan University of Medicine, Huaihua, China; ^3^ Bioland Laboratory (Guangzhou Regenerative Medicine and Health Guangdong Laboratory), Guangzhou, China

**Keywords:** *Arnebiae Radix*, estrogen receptor, affinity ultrafiltration, molecular docking, UHPLC-Q-Exactive Orbitrap mass spectrometry, breast cancer

## Abstract

*Arnebiae* Radix (dried root of *Arnebia euchroma* (Royle) Johnst.) is a traditional Chinese medicine (TCM) used to treat macular eruptions, measles, sore throat, carbuncles, burns, skin ulcers, and inflammations. The *Arnebiae* Radix extract can exert anti-breast cancer effects through various mechanisms of action. This study aimed to rapidly screen potential estrogen receptor (estrogen receptor α and estrogen receptor β) ligands from the *Arnebiae* Radix extract. In this study, an analytical method based on affinity ultrafiltration coupled with UHPLC-Q-Exactive Orbitrap mass spectrometry was established for rapidly screening and identifying estrogen receptor ligands. Then, bindings of the components to the active site of estrogen receptor (estrogen receptor α and estrogen receptor β) were investigated *via* molecular docking. Moreover, surface plasmon resonance (SPR) experiments with six compounds were performed to verify the affinity. As a result, a total of 21 ligands were screened from *Arnebiae* Radix using affinity ultrafiltration. Among them, 14 and 10 compounds from Arnebiae Radix showed affinity with estrogen receptor α and estrogen receptor β, respectively. All of those ligands could have a good affinity for the multiple amino acid residues of the estrogen receptor based on molecular docking. In addition, six compounds display the great affinity by SPR. The method established in the study could be used to rapidly screen estrogen receptor ligands in Traditional Chinese medicine. The results demonstrated that the affinity ultrafiltration–UHPLC-Q-Exactive Orbitrap mass spectrometry method not only aids in the interpretation of the potential bioactive components and possible mechanisms of action of *Arnebiae Radix* but also provides a further effective basis for the quality control of this valuable herb medicine.

## Graphical Abstract

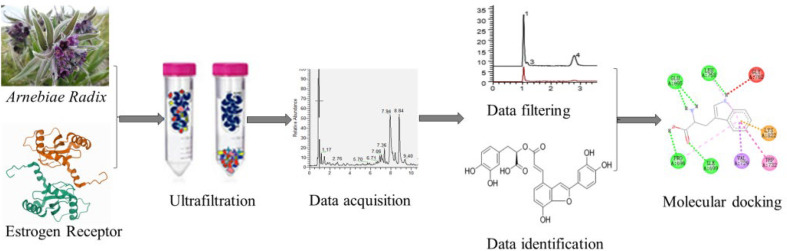

## Introduction

Breast cancer is a hormone-dependent tumor, and its incidence ranks first among female malignant tumors, which seriously endangers women’s physical and mental health (WHO, 2022). It is currently believed that estrogen receptors (ER α and ER β) are closely related to the occurrence and development of breast cancer (Liu and Zheng, 2011; Pelekanou et al., 2012; Qi et al., 2012). Clinically, breast cancer hormone replacement therapy (HRT) is based on ER as a treatment index (Larionov and Miller, 2009), such as tamoxifen and fulvestrant which are ER-targeted drugs for the treatment of breast cancer (William and Kilian, 2016; Li et al., 2018), but there are problems such as large adverse reactions and insignificant therapeutic effects (Zhou et al., 2021). Considering these side effects, the active ingredients in medicinal plants have attracted widespread attention due to their biological activities and low toxicity. Plants have been used for medicinal purposes for centuries. They also include active ingredients for human health (Dogan et al., 2014; Saran et al., 2021; Tamer et al., 2021). Therefore, the development and clinical application of targeted drugs for the signal pathways related to the occurrence and development of breast cancer have become a new hot spot in breast cancer treatment research (Walker et al., 2015), which also opens up new ideas for the clinical treatment of advanced or metastatic breast cancer.

Recently, WHO (World Health Organization) estimated that 80% of people worldwide rely on herbal medicines for some aspects of their primary healthcare needs. According to WHO, around 21,000 plant species have the potential for being used as medicinal plants (WHO, 2022). *Arnebiae* Radix is the dry root of *Arnebia euchroma* (Royle) Johnst. It is mainly distributed in Xinjiang, Mongolia, and Northeast China (Zhang et al., 2019). *Arnebiae* Radix is a commonly used Chinese medicine in clinical practice, and it is often used to treat diseases such as chronic hepatitis and liver cirrhosis (Zhu et al., 2021). A number of studies have found that *Arnebiae* Radix has significant antioxidant, anti-inflammatory, antibacterial, and anticancer pharmacological effects (Wang et al., 2019) and has been proved to have a good effect on many refractory diseases such as cancer, AIDS, allergic purpura, and psoriasis (Zhu, 2013). In addition, folks often use *Arnebiae* Radix as a raw material for the treatment of breast cancer (Deng, 2019). Studies have shown that the anticancer effects of naphthoquinone compounds, the main active components of *Arnebiae* Radix, have also been extensively studied. For example, shikonin can inhibit the proliferation of esophageal cancer (Tang et al., 2018), lung cancer (Li et al., 2017; Hsieh et al., 2017), glioblastoma (Zhao et al., 2015), breast cancer (Yang et al., 2019), etc. Moreover, the *Arnebiae* Radix extract can exert anti-breast cancer effects through various mechanisms of action (Chen et al., 2019; Lin et al., 2020; Yan et al., 2021). Shikonin and 4-hydroxytamoxifen synergistically inhibit the proliferation of breast cancer cells by activating the apoptosis signaling pathway *in vitro* and *in vivo* (Lin et al., 2020), and some studies have reported that natural products such as “shikonin” may be the hope for the treatment of breast cancer (Khan and Maidul, 2019). It can be seen that *Arnebiae* Radix has a certain curative effect in the treatment of breast cancer. However, the mining of the anticancer active ingredients of *Arnebiae* Radix is not sufficient, so it is necessary to conduct a more in-depth study on the anticancer material basis of *Arnebiae* Radix.

Based on the complexity of chemical components and the trace level of biologically active ingredients, screening potential targeted ligands from natural drugs is a challenging task (Wu et al., 2016). Compared with traditional screening methods, the affinity ultrafiltration method has some inherent advantages, including quickness, simple operation, and being target-oriented (Wei et al., 2016). The method is established depending on the specificity of biological affinities between ligands and enzymes. As a fast and high-throughput screening technology, affinity ultrafiltration is divided into four steps: incubation, separation, dissociation, and analysis by LC/MS (Qin et al., 2015; Zhang et al., 2017; Long et al., 2020; Yuan and Hu, 2021). In recent years, affinity ultrafiltration-mass spectrometry (AUF-MS) technology has been successfully used in the screening of lead compounds and active ingredients of natural products. Liu et al. (2013) combined ultrafiltration screening and used UPLC-DAD-ESI-MS^n^ technology to find 12 substances with XOD inhibitory effects from the extract of *Salvia miltiorrhiza* Bge.

Surface plasmon resonance (SPR) is a kind of biochemical detection technology based on physical optical phenomena, which is an advanced method to study biomolecular interactions due to its real-time detection, no marking, and strong anti-interference (Olaru et al., 2015). For example, Wang et al. using SPR demonstrated that berberine directly interacted with thrombin with a K_D_ value of 16.39 M (Wang et al., 2017).

In the present study, two target molecules (ER α and ER β) were carefully picked out for the screening of anticancer compounds from *Arnebiae* Radix based on the pathogenesis of breast cancer and its current status of drug development. This work introduced AUF-MS into screening active compounds in *Arnebiae* Radix extracts at a molecular level. Furthermore, the bioactivity of *Arnebiae* Radix and its components were validated by molecular docking. This work also explored the pharmacodynamic material basis of *Arnebiae* Radix anti-breast cancer according to the relationship of compound–target.

## Materials and methods

### Chemicals and reagents

ER α, ER β, and buffer recombinant protein were purchased from Shanghai Tongwei Biotechnology Co., Ltd. (China). Ultrafiltration membranes (0.5 ml, 10 kDa) were purchased from Millipore Co., Ltd. (USA).

The MS-grade formic acid and acetonitrile were purchased from Thermo Fisher Scientific Co., Ltd. (USA). The ultra-pure water was obtained from Guangzhou Watsons Food & Beverage Co., Ltd. (China). Dimethyl sulfoxide (DMSO, W387520) was purchased from Sigma-Aldrich Chemical Co. (St. Louis, MO, USA). Other solvents were of analytical grade supplied by Aladdin Industrial Corporation. The chemical reference standards of malic acid, citric acid, caffeic acid, salvianolic acid B, rosmarinic acid, and salvianolic acid C were purchased from Chengdu Pufei De Biotech Co., Ltd. (China). Rutin, isoquercitrin, and quercetin were provided by Cheng Du Herbpurify Co., Ltd. (China). The purities of all the reference standards were above 98% according to HPLC-UV analysis.

### Sample preparation

*Arnebiae* Radix was obtained from Xinjiang Zhaosu County and authenticated by Professor Shengjun Ma. The voucher specimens were deposited at the School of Pharmaceutical Sciences, Hunan University of Medicine.

Initially, *Arnebiae* Radix herbs were ground into powder before sample preparation and sieved through a No. 40 mesh. Then, the dried powder of *Arnebiae* Radix (10 g) was sonication-extracted in 200 ml methanol for 1 h at room temperature, and the extracted solution was filtered and dried by rotary evaporation. Finally, the concentrate was dried in a freeze vacuum drying oven. These samples were collected and stored in a desiccator for subsequent ER affinity ultrafiltration screening experiments.

### Affinity ultrafiltration screening

The above extract was dissolved in DMSO, and the solution was diluted with buffer (pH = 8.0) to a final concentration of 5.0 mg/ml. The sample solution and buffer were successively added to EP tubes. Then buffer, inactivated ER α solution (200 µg/ml, boiled in the water bath for 20 min), and ER α solution (200 µg/ml) were separately added as the blank control group, the denatured control group, and the sample group, respectively. The blank control group and denatured control group were set to exclude some interference factors about background, false positives, and non-specific binding. The group of mixtures was subsequently incubated in the dark at 37°C for 60 min to make a combination balance. Next, each mixture (200 μl) was transferred into an ultrafiltration tube and ultrafiltered at 12,000 rpm for 15 min at 4°C. Then, each group was washed three times with 300 μl of buffer to remove unbound small molecules. Finally, the ligands bound to ER α were dissociated from the complexes by incubating with 300 μl of methanol/water (90/10) for 10 min and then centrifuging at 12,000 rpm for 15 min at room temperature, three times. These ultrafiltrates for each group were dried under nitrogen gas at room temperature and reconstituted in 80 μl of methanol/water (50/50) prior to UHPLC-Q-Exactive Orbitrap MS analysis. All the screening assays were carried out in triplicate. Except for the replacement of the ER, the affinity ultrafiltration screening method of ER β is similar to that of ER α in the other steps.

### UHPLC-Q-Exactive orbitrap MS analysis

The analyses are exercised on Q-Exactive Focus Orbitrap MS connected to Thermo Scientific Dionex Ultimate 3000 RS through an ESI source. For the chromatographic separation, a Thermo Scientific Hypersil GOLD™ aQ (100 mm × 2.1 mm, 1.9 μm) was applied at the flow rate of 0.3 ml/min. The 0.1% formic acid and acetonitrile were used as the mobile phases A and B, respectively. The optimized UHPLC elution procedures were conducted as follows: 0–2 min, 95%–90% A; 2–5 min, 90%–80% A; 5–10 min, 80%–75% A; 10–15 min, 75%–50% A; 15–25 min, 50%–45% A; 25–40 min, 45%–20% A; 40–45 min, 20%–5% A; 45–45.1 min, 5%–95% A; 45–50 min, 95% A. The column temperature was maintained at 35°C. For the MS/MS analysis, the mass spectrometer equipped with an electrospray ionization source was obtained with a resolution of 70,000 detected by the Orbitrap analyzer. In order to comprehensively explore compounds, the positive and negative ion modes were both selected. The mass spectrometry conditions are as follows: electrospray ion source, the spray voltage was 3.5 kV for positive ion mode and 3.2 kV for negative ion mode, sheath gas pressure was 35 arb, auxiliary gas pressure was 10 arb, and the temperature of capillary and auxiliary gas heater was 320°C and 350°C, respectively; and the S-lens RF level was 60. Full-scan data were acquired from *m/z* 100–1,200 by data-dependent MS^2^ scanning or parallel reaction monitoring (PRM) mode. The nitrogen gas served as collision gas, and the energy was set as normalized collision energy 30%. Instrument and data were controlled by the Xcalibur software version 4.2.

### Molecular docking

To explore how the 21 screened compounds with strong binding activity conjugate with ER α/ER β, the binding interactions between compounds and ER α/ER β were researched by molecular docking. The estradiol was used as a positive control. The chemical structures of the core active ingredients were downloaded from the PubChem database, hydrotreated with Discovery Studio 2018 software, and converted into pdb format. The protein crystal structure was downloaded from the Protein Data Bank (PDB) database (https://www.rcsb.org/), “Homo sapiens” was selected as the species, and the resolution was A ≤3. The three-dimensional structure of ER α (PDB ID: 1A28) and ER β (PDB ID: 1X7B) was acquired from the PDB. The protein crystals were imported into Discovery Studio software, water molecules were removed, and polar hydrogen atoms were added, and after treatment with a force field, they were saved in pdb format. AutoDock was used to convert the processed protein molecule into PDBQT format and determine the binding site; SailVina software was used for molecular docking, and the binding ability between the compound and the target protein was evaluated according to the docking binding affinity (affinity, kcal/mol). Discovery Studio software visualized the results. Docking and scoring were performed with Schrodinger Suites (version 2019-1).

### SPR measurements

Six of the compounds screened by AUF-LC-MS were selected for further SPR experiments to verify the binding to ER β, including rosmarinic acid, quercetin, salvianolic acid C, rutin, isoquercitrin, and salvianolic acid B.

SPR binding measurements were performed using a Biacore T200 biosensor system (Cytiva, USA). All the SPR-based materials were acquired from Cytiva.

ER β was dissolved in PBS at 1.0 mg/ml and was then diluted in 20 μg/ml sodium acetate buffer at pH 5.0 and immobilized on a CM5 chip using an amine coupling kit. Sulfo-NHS (0.1 mol/l) and EDC (0.4 mol/l) with a 1:1 volume ratio were mixed at 25°C and flowed through a chip channel at 10 μl/min. ERβ was flowed through the chip channel at the same flow rate and reacted with the activated carboxyl groups on the chip for 600 s. Ethanolamine hydrochloride flowed through the chip channel at the same flow rate for 360 s.

Sensor preparation and interaction analyses were performed at 25°C in the PBS running buffer. Each analyte was dissolved in DMSO at 1 mg/ml and then dissolved in the running buffer at 0.20, 0.39, 0.78, 1.56, 3.13, 6.25, 12.5, and 25 μg/ml for SPR measurements.

The running buffer was used as the blank control. Eight groups of gradient concentrations of the same analyte (and the blank control) were flowed through the immobilized channel at a flow rate of 30 μl/min, with an association time of 180s and a dissociation time of 300 s. The collected signals were monitored in real time, and a reference flow cell without immobilized ER β served as a non-specific binding control. The rate constants of association and dissociation were calculated by the non-linear fitting method including the simultaneous fitting for the association and dissociation phases in each sensorgram in Biacore Evaluation Software version 3.2.1 (Cytiva, USA).

### Statistical analysis

All of the results were expressed as the mean ± standard deviations (n = 3). The significance was analyzed using one-way analysis of variance (ANOVA), and the difference was considered to be statistically significant at p < 0.05. Statistical analysis was performed using GraphPad Prism 5.

## Results and discussion

### Optimization of AUF-LC-MS screening

Relevant literature and preliminary experiments show that the binding between specific ligands and receptors is crucial for affinity experiments. Therefore, different *Arnebiae* Radix extract concentrations (5 and 10 mg·ml^-1^), ER concentrations (200 and 400 μg/ml), and incubation times (30, 60, and 90 min) were investigated during the incubation experiment. Finally, 5 mg·ml^-1^ of *Arnebiae* Radix and 200 μg/ml of ER were chosen based on the number and intensity of the peak in UHPLC-Q-Exactive Orbitrap MS.

### Identification of ER α/ER β ligands from *Arnebiae* Radix

MS analysis was performed with ESI in negative and positive ion modes owing to a stronger response in mass spectrometry. Retention time (Rt), calculated and measured molecular mass, mass error, MS/MS fragment ions, molecular formula, and identification results are summarized in **Table 1**. Moreover, the standards of malic acid, citric acid, caffeic acid, rutin, quercetin, isoquercitrin, salvianolic acid B, rosmarinic acid, and salvianolic acid C were used to verify the accuracy of compound identification. Eventually, a total of 21 compounds were identified by matching fragment ions in references (Zhu et al., 2022) or by searching in the databases such as chemical structure database (ChemSpider) and MS^2^ database (mzVault and mzCloud).

**Table 1:** AUF-LC-MS data for possible ER α/ER β ligands in *Arnebiae Radix*.

No.	tR	Theoretical Mass m/z	Experimental Mass m/z	Error (ppm)	Formula	MS/MS fragment(-)	MS/MS fragment(+)	Identification	Specific binding
1	0.97^*^	133.01424	133.01303	0.95	C_4_H_6_O_5_	MS^2^[133]: 115.0024(100), 71.0124(42), 133.0130(34)		Malic acid^b^	2.20
2	1.14^*^	191.01972	191.01917	-2.91	C_6_H_8_O_7_	MS^2^[191]: 111.0074(100), 87.0074(33), 85.0281(21), 191.0187(12)		Citric acid^a,b^	5.02^a^ 1.78^b^
3	1.34	132.10190	132.10220	2.24	C_6_H_13_NO_2_		MS^2^[132]: 86.0971(100), 132.1022(3)	Leucine^a^	5.26
4	1.35	132.10190	132.10179	-0.88	C_6_H_13_NO_2_		MS^2^[132]: 86.0967(100)	Norleucine^b^	2.29
5	1.94	166.08625	166.08652	1.60	C_9_H_11_NO_2_		MS^2^[166]: 120.0811(100), 84.9603(25), 166.0865(7)	Phenylalanine^a,b^	6.36^a^ 2.39^b^
6	2.80	145.05063	145.04974	1.66	C_6_H_10_O_4_	MS^2^[145]: 101.0596(100), 83.0490(92), 145.0497(51)		Adipic acid^a^	5.77
7	3.48	205.09715	205.09746	1.50	C_11_H_12_N_2_O_2_		MS^2^[205]: 188.0708(100), 146.0602(47)	Tryptophan^a^	4.19
8	4.33	165.01933	165.01851	1.95	C_8_H_6_O_4_	MS^2^[165]: 121.0284(100), 136.9311(45), 96.9588(19), 165.0185(13)		Terephthalic acid^a^	1.83
9	5.22^*^	179.03498	179.03433	-3.65	C_9_H_8_O_4_	MS^2^[179]:135.0441(100), 179.0343(37), 150.9531(10)		Caffeic acid^a^	1.61
10	6.96	537.10384	537.10474	1.66	C_27_H_22_O_12_	MS^2^[537]: 197.0450(100), 339.0511(84), 135.0441(73), 295.0613(65), 229.0140(40), 179.0343(40)		Salvianolic acid J^a^	1.86
11	7.56^*^	609.14610	609.14697	1.42	C_27_H_30_O_16_	MS^2^[609]: 300.0277(100), 301.0351(56)		Rutin^a,b^	1.80^a^ 1.76^b^
12	7.57^*^	303.04992	303.04916	-2.54	C_15_H_10_O_7_		MS^2^[303]: 303.0490(100), 304.0525(11), 257.1164(7), 199.0385(7), 239.1057(2)	Quercetin^b^	1.54
13	7.83^*^	463.08819	463.08871	1.11	C_21_H_20_O_12_	MS^2^[463]: 300.0273(100), 301.0346(53), 151.0025(4), 463.0881(4), 178.9980(4)		Isoquercitrin^b^	1.91
14	8.08^*^	717.14610	717.14709	1.37	C_36_H_30_O_16_	MS^2^[717]: 339.0509(100), 321.0759(36), 109.0282(34), 197.0448(30), 295.0606(21), 321.0400(7),		Salvianolic acid B^b^	2.24
15	8.95^*^	359.07724	359.07700	-0.67	C_18_H_16_O_8_	MS^2^[359]: 161.0234(100), 197.0447(35), 179.0340(16), 72.9917(11)		Rosmarinic acid^b^	2.66
16	10.73	273.11213	273.11227	0.50	C_16_H_16_O_4_		MS^2^[273]: 203.0340(100), 208.9551(70), 151.0390(54), 255.1014(37), 217.0493(35), 90.0554(32)	Deoxyshikonin^a^	2.28
17	11.08	343.11871	343.11676	-2.94	C_19_H_20_O_6_	MS^2^[343]: 253.1207(100), 343.1165(73), 297.1110(69), 255.1372(21), 161.0234(15)		1-methoxyacetylshikonin^a^	1.95
18	11.08	287.12778	287.12808	1.03	C_17_H_18_O_4_		MS^2^[287]: 199.0392(100), 269.1173(78), 241.1223(38), 251.1068(26), 287.1280(24)	Sativan^a^	2.15
19	11.14	201.11323	201.11269	-2.70	C_10_H_18_O_4_	MS^2^[201]: 164.8354(100), 201.1128(69), 139.1118(62), 166.8324(29), 183.1021(29), 112.9387(14)		Sebacicacid^a^	2.96
20	11.85	554.09720	554.09485	0.02	C_41_H_15_O_3_	MS^2^[554]: 311.0563(100), 135.0441(22), 61.9870(5), 197.0450(4)		Salvianolic acid^a^	1.53
21	11.99^*^	491.09837	491.09808	-0.59	C_26_H_20_O_10_	MS^2^[491]: 135.0440(100), 197.0448(12), 179.0340(9), 72.9916(5)		Salvianolic acid C^b^	1.75

^*^ Identified by comparison with standards.

^a^ and ^b^ represented the compound was present in the ER α and ER β ultrafiltration, respectively.

**Figure 1:**
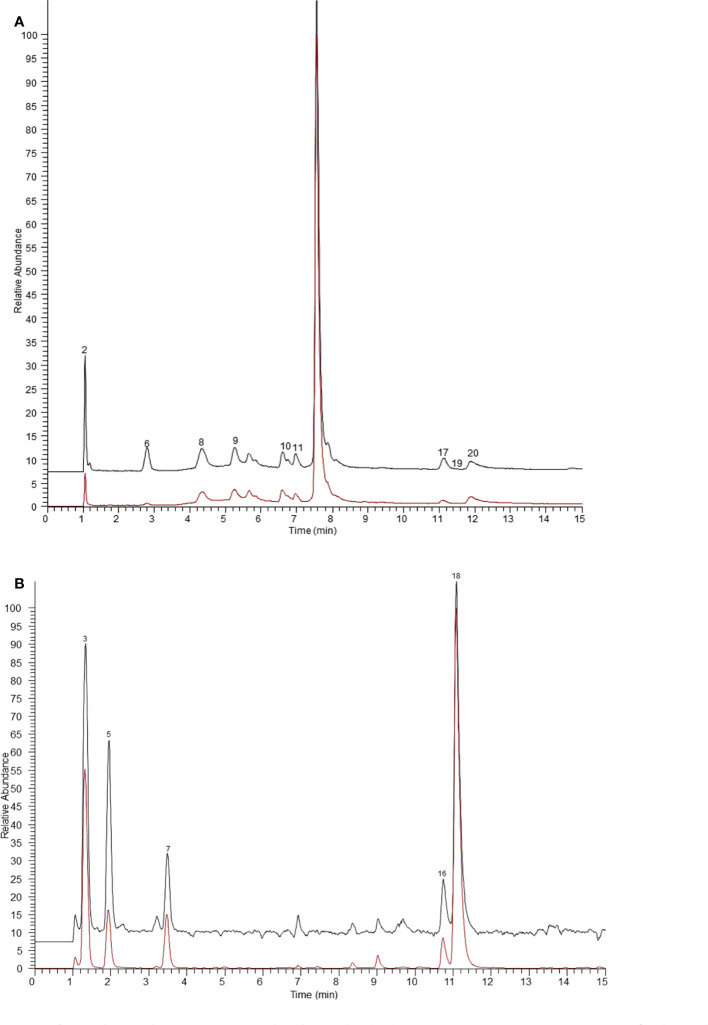
The ultrafiltration UHPLC-Q-Exactive Orbitrap MS chromatograms of the potential ER α ligands in *Arnebiae Radix*. **(A)** Extracted ion chromatograms of compounds bound to ER α in negative ion mode. **(B)** Extracted ion chromatograms of compounds bound to ER α in positive ion mode. The black line represents active ER α group, the red line represents denatured ER α group.

**Figure 2:**
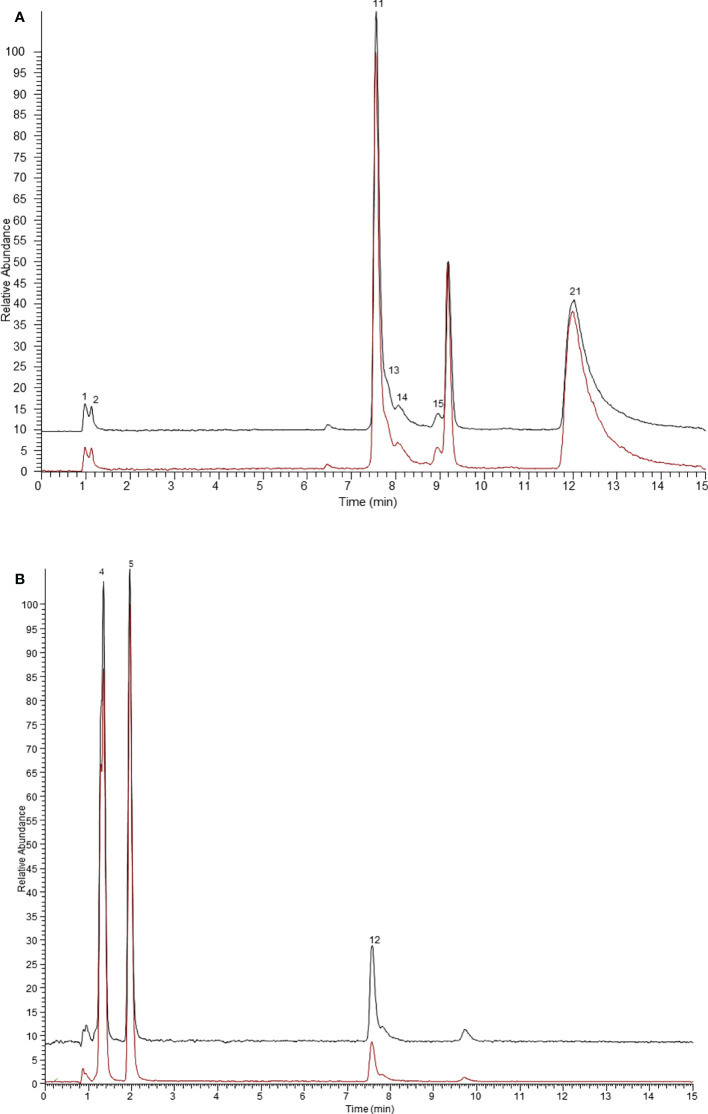
The ultrafiltration UHPLC-Q-Exactive Orbitrap MS chromatograms of the potential ER β ligands in *Arnebiae Radix*. **(A)** Extracted ion chromatograms of compounds bound to ER β in negative ion mode. **(B)** Extracted ion chromatograms of compounds bound to ER β in positive ion mode. The black line represents active ER β group, the red line represents denatured ER β group.

In general, the peak area ratios of components with higher affinity for the target enzyme are significantly higher than those of components with lower or no affinity (Tao et al., 2015). Therefore, affinity ultrafiltration can rapidly screen and identify bioactive compounds in complex plant extracts by comparing the changes in peak areas before and after ultrafiltration. As shown in **Figures 1** and **2**, it is easy to recognize compounds exhibiting specific bindings to ER α/ER β from *Arnebiae* Radix according to the UHPLC-Q-Exactive Orbitrap MS chromatograms. By comparing the peak area of the ultrafiltrate between active and denatured groups, specific binding to ER α/ER β was defined to rank the binding affinity using the following formula: specific binding = A_a_/A_b_, where A_a_ and A_b_ are the relative peak areas of compounds in the active group and denatured group, respectively (Xie et al., 2020). According to literature reports (Munigunti et al., 2011), compounds whose specific binding values were less than 1.5 were considered as non-ligands.

As shown in **Figure 1**, after affinity ultrafiltration screening, 14 components from *Arnebiae* Radix displayed that they could bind to ER α; the specific binding of each component is exhibited in **Table 1**. Among them, phenylalanine possessed the greatest specific binding (6.36), followed by adipic acid (5.77), leucine (5.26), citric acid (5.02), tryptophan (4.19), sebacic acid (2.96), deoxyshikonin (2.28), sativan (2.15), salvianolic acid J (1.86), terephthalic acid (1.83), rutin (1.80), caffeic acid (1.61), and salvianolic acid (1.53). Therefore, 14 compounds were considered as potential ER α ligands, which displayed the binding affinity >1.5.

Similarly, the results of the binding capacities of ligands in *Arnebiae* Radix ultrafiltrate to ER β are shown in **Figure 2**. According to the retention time and mass spectrometry information of each chemical component, 10 compounds were screened out as the potential ER β ligands because of their high specific binding value which all exceeded 1.5. Among them, rosmarinic acid possessed the greatest specific binding (2.66). The specific binding ability of other compounds is shown in **Table 1**.

The screened compounds mainly include organic acids, flavonoids, shikonins, and amino acids. Organic acid active compounds in *Arnebiae* Radix mainly include caffeic acid, salvianolic acid B, rosmarinic acid, and salvianolic acid C. They have anticancer (Gomes et al., 2003) and anti-malignant cell proliferation (Soo, 2005) biological activities. Salvianolic acid compounds are a potential source of natural medicine for cancer treatment (Ma et al., 2019), among which the potential medicinal activity of salvianolic acid B is to fight cancer by targeting multiple signaling pathways (Qin et al., 2019). Flavonoid active ingredients such as rutin, quercetin, sativan, and isoquercitrin have various pharmacological effects such as antiviral, anti-inflammatory, and anticancer (Ozçelik et al., 2011). Among them, rutin is a safe anticancer drug with few side effects. In recent years, studies on the anticancer mechanism of this natural drug *in vivo* and *in vitro* have been widely carried out (Imani et al., 2020). Mohammed et al. (Mohammed et al., 2021) studied the apoptosis, gene expression, and cytotoxicity of quercetin on MCF-7 breast cancer cell lines and preliminarily established the *in vitro* efficacy and *in vivo* safety of quercetin as a potential anti-breast cancer formula. In addition, the shikonin components in *Arnebiae* Radix have significant anticancer effects (Zhu et al., 2021), and deoxyshikonin, as a leading compound, also shows good anticancer effects through structural modification (Ma et al., 2021). The reliability of the affinity ultrafiltration screening method was further demonstrated.

### Molecular docking analysis

In this study, the molecular docking method was used to predict the binding mode and binding affinity of the potential ER α/ER β ligands screened by AUF-LC-MS to the amino acids associated with the corresponding receptor binding sites. Among them, the binding affinity of ligands and receptors is shown in **Table 2**.

**Table 2:** Estrogen receptor ligands binding energy.

Compounds	ER α ligands Binding energy (kcal/mol)	ER β ligands Binding energy (kcal/mol)
Sativan Deoxyshikonin 1-methoxyacetylshikonin Tryptophan Salvianolic acid J Salvianolic acid Rutin Caffeic acid Phenylalanine Terephthalic acid Sebacicacid Citric acid Adipic acid Leucine	-9.3 -8.5 -7.9 -7.1 -7.1 -6.7 -6.5 -6.3 -6.2 -5.9 -5.8 -5.7 -5 -4.8	/ / / / / / -6.4 / -6 / / -5.7 / /
Rosmarinic acid Quercetin Salvianolic acid C Isoquercitrin Salvianolic acid B Malic acid Norleucine	/ / / / / / /	-8.4 -8 -7.1 -6.3 -6 -5.2 -5.1

**Figure 3:**
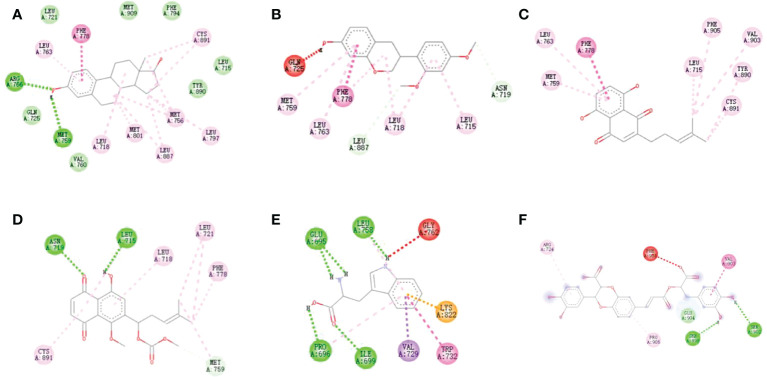
Docking models of **(A)** estradiol, **(B)** Sativan, **(C)** Deoxyshikonin, **(D)** 1-methoxyacetylshikonin, **(E)** Tryptophan and **(F)** Salvianolic acid J in ER α active site. (Hydrogen bonds were colored in green; hydrophobic interactions were colored in red).

**Figure 4:**
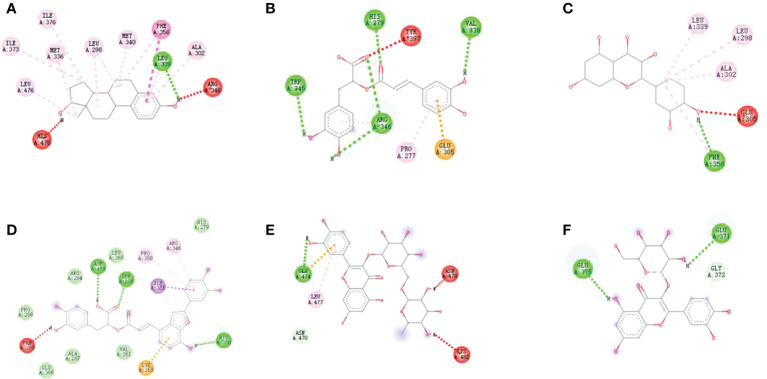
Docking models of **(A)** Rosmarinic acid, **(B)** Quercetin, **(C)** Salvianolic acid C, **(D)**Rutin, **(E)** Isoquercitrin and **(F)** Phenylalanine in ER β active site. (Hydrogen bonds were colored in green; hydrophobic interactions were colored in red).

The binding energy of positive compound estradiol was -10.3/-10.6 kcal/mol, indicating that estradiol and ER α/ER β had a strong interaction. Additionally, an ER α-specific region mainly consists of the amino acids Leu 715, Phe 778, Met 759, Leu 763, Leu 718, and Cys 891. Thereby, it could be observed from **Figures 3** and **4**, which proved that the protein active pocket established in this experiment was reasonable. Furthermore, sativan (binding energy = -9.3 kcal/mol) displayed the largest binding interactions with the ER α binding site, followed by deoxyshikonin (-8.5 kcal/mol), 1-methoxyacetylshikonin (-7.9 kcal/mol), tryptophan (-7.1 kcal/mol), and salvianolic acid J (-7.1 kcal/mol).

The binding energy of estradiol was -10.6 kcal/mol. The interactions registered by estradiol with the ER β binding pocket were as shown in **Figure 4**. The binding energies calculated for the 10 compounds with ER β were in the range of -5.1 to -8.4 kcal/mol. The best binding energy was exhibited by rosmarinic acid (-8.4 kcal/mol). All the implications from the molecular docking study further confirm the reliability of the ultrafiltration screening method.

### SPR-based binding studies

The immobilized level of ER β was 6370 RU (response unit), and the occupancy of ER β on the surface of sensor chip was 6.37 ng/mm^2^, which was suitable for interaction studies with small molecules.

**Figure 5:**
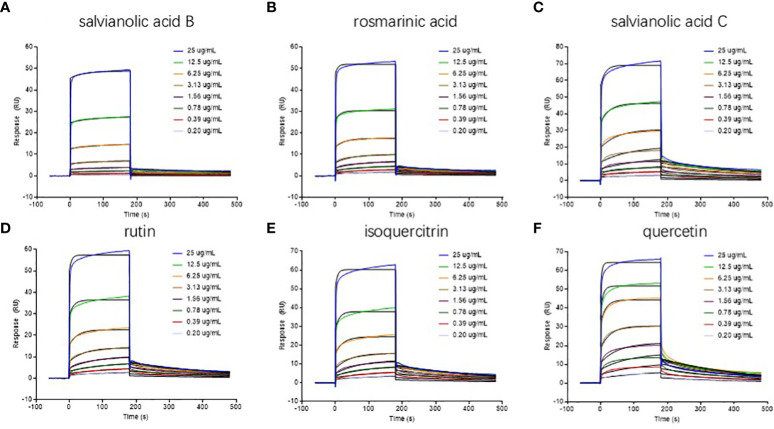
Real-time sensorgrams of kinetic analysis of the interactions between drugs and ERβ at 25 °C **(A)** salvianolic acid B; **(B)** rosmarinic acid; **(C)** salvianolic acid C; **(D)** rutin; **(E)** isoquercitrin; **(F)** quercetin.

The binding patterns of six analytes to ER β are shown in **Figure 5**. The kinetic parameters obtained are summarized in **Table 3**. The association rate constant (*K_a_*) and dissociation rate constant (*K_d_*) are parameters that express the formation rate of complex and the stability of complex, respectively. *K_D_* reflects the binding capacity or affinity; the greater the affinity, the smaller the *K_D_*. The results show that the *K_a_*, *K_d_*, and *K_D_* of analytes with ER β were 10^2^~10^3^, 10^-3^, and 10^-6^ M levels, respectively, suggesting that these six compounds could bind well with ER β. For example, rutin had a relatively high affinity with a *K_D_* value of 9.34×10^-7^ M. The SPR study confirmed that six drugs screened by AUF-LC-MS did have an ER β binding activity, which further confirmed the reliability of the ultrafiltration screening method and molecular docking.

**Table 3:** Kinetic parameters of drugs with ERβ.

Drug	*K_a_* (M^-1^s^-1^)	*K_d_* (s^-1^)	*K_D_* (M)	*R_max_* (RU)	Chi^2^	U-value
salvianolic acid B	7.88×10^2^	1.83×10^-3^	2.32×10^-6^	3.51	0.17	8.96
rosmarinic acid	2.02×10^3^	2.66×10^-3^	1.32×10^-6^	4.41	0.24	6.89
salvianolic acid C	1.39×10^3^	2.76×10^-3^	1.98×10^-6^	12.6	0.62	4.08
rutin	3.70×10^3^	3.46×10^-3^	9.34×10^-7^	7.78	0.42	5.3
isoquercitrin	3.02×10^3^	3.14×10^-3^	1.04×10^-6^	9.57	0.62	5.3
quercetin	2.95×10^3^	3.72×10^-3^	1.26×10^-6^	13.28	1.01	5.3

## Conclusions

In the present study, a convenient, rapid, and accurate AUF-LC-MS strategy with multiple drug targets of ER α/ER β was developed to explore the pharmacodynamic material basis components of *Arnebiae* Radix anticancer in a multicomponent and multitarget manner. As a result, 14 and 10 compounds from *Arnebiae* Radix showed affinity with ER α/ER β, respectively. Then, the molecular docking assay explained the relationship between structure and function at the molecular level. Moreover, six of the compounds screened by AUF-LC-MS and molecular docking were selected for further SPR to verify the great affinity to ER β. Overall, the affinity ultrafiltration LC-MS successfully screened out multiple potential ER ligands from the *Arnebiae* Radix extract, which will not only aid in the interpretation of the potential bioactive components and possible mechanisms of action of *Arnebiae* Radix but also provide a further effective basis for the quality control of this valuable herb medicine.

## Data availability statement

The original contributions presented in the study are included in the article/supplementary material. Further inquiries can be directed to the corresponding authors.

## Author contributions

LZ and S-jM contributed equally; LZ: writing—original draft, performed experiments, and sorted the data; S-jM: conceptualization, analyzed the validation data; M-jL: data curation; K-lL, SE, Z-mW, and S-nL: performed the experiments and analyzed the data; S-l Z: performed the SPR experiments; WC: writing—review and editing, conceptualization, supervision. All data were generated in-house, and no paper mill was used. All authors agree to be accountable for all aspects of work ensuring integrity and accuracy. All authors contributed to the article and approved the submitted version.

## Funding

This work was supported by the Natural Science Foundation of Xinjiang Uygur Autonomous Region (2020D03002) and Graduate Student Innovation Project in Xinjiang Uygur Autonomous Region (XJ2021G175).

## Conflict of interest

The authors declare that the research was conducted in the absence of any commercial or financial relationships that could be construed as a potential conflict of interest.

## Publisher’s note

All claims expressed in this article are solely those of the authors and do not necessarily represent those of their affiliated organizations, or those of the publisher, the editors and the reviewers. Any product that may be evaluated in this article, or claim that may be made by its manufacturer, is not guaranteed or endorsed by the publisher.
